# Plant assisted synthesis of silver nanoparticles using *Persicaria perfoliata (L.)* for antioxidant, antibacterial, and anticancer properties

**DOI:** 10.1016/j.heliyon.2024.e40543

**Published:** 2024-11-21

**Authors:** Deepak Kumar Shrestha, Dipak Raj Jaishi, Indra Ojha, Dinesh Raj Ojha, Ishwor Pathak, Akash Budha Magar, Niranjan Parajuli, Khaga Raj Sharma

**Affiliations:** a Central Department of Chemistry, Tribhuvan University, Kirtipur, Kathmandu, Nepal; b Department of Chemistry, Butwal Multiple Campus, Tribhuvan University, Nepal; c Department of Chemistry, Amrit Campus, Tribhuvan University, Nepal

**Keywords:** Antidiabetic, Antimicrobial, Antioxidant, Cytotoxicity, Green synthesis, *Persicaria perfoliata*, Silver nanoparticles

## Abstract

*Persicaria perfoliata* (L.) is an herbaceous medicinal plant belonging to the Polygonaceae family. The plant is distributed in Nepal, India, Japan, China, Russia, and Korea. The present study involved the analysis of plant secondary metabolites, synthesis of silver nanoparticles (Ag NPs) using the plant, characterization, and exploration of antioxidant, antidiabetic, antibacterial, and cytotoxic activities. Among six different solvent extracts, the methanol extract displayed the highest total phenolic content (TPC) and total flavonoid content (TFC) of 68.61 ± 0.57 mg GAE/g and 40.69 ± 5.0 mg QE/g respectively. Ag NPs and hexane extract displayed the potential antioxidant activity of IC_50_ 69.40 ± 0.13 and 144.50 ± 1.36 μg/mL in the DPPH assay. The α-amylase inhibition shown by an aqueous extract and the synthesized Ag NPs IC_50_ of 1188.83 ± 33.52 and 1369.30 ± 46.86 μg/mL respectively. In antibacterial activity, the highest ZOI of 16 mm was displayed by Ag NPs against *Klebsiella pneumoniae* followed by a ZOI of 11 mm for methanol extract against *Shigella sonnei*. Similarly, the lowest MIC and MBC of 0.78125 and 1.5625 mg/mL were recorded for both Ag NPs and methanol extract against *Staphylococcus aureus*. Aqueous extract and Ag NPs did not display significant toxicity against brine shrimp nauplii. Ag NPs displayed an IC_50_ of 251.86 ± 58.90 μg/mL against HeLa cell lines. Biosynthesized Ag NPs showed a distinct peak at 409 nm in UV–visible spectra. FTIR analysis revealed the involvement of different functional groups of the organic compounds present in plant extract as reducing, capping, and stabilizing agents in the synthesis of Ag NPs. XRD analysis confirmed the crystal structure of Ag NPs, whereas the average grain size of 44.28 nm was determined by FE-SEM analysis. EDX spectra established the elemental composition of Ag NPs. The present study shows the synthesized Ag NPs using plant extract impart the potential biological activities as compared to that of the crude extract.

## 1 Introduction

Medicinally high-value plant species are beneficial for treating illnesses and enhancing human health [1]. These plants have been used in traditional medical systems to cure various diseases. Specific examples include the use of *Bryonia alba, Tinospora cordifolia, and Calotropis gigantea,* for the treatment of pneumonia, jaundice, and asthma respectively [2]. According to WHO, around 80 % of the world's population gets health benefits from traditional medicinal practices [3]. *Persicaria perfoliata* (L.) is an important medicinal plant widely used in Chinese traditional medicine. It is an annual herbaceous plant belonging to the Polygonaceae family. The plant is an invasive climbing/twinning vine. It is found in China, North Korea, Japan, Indonesia, Philippines, India, Nepal and Russia. It has been utilized as traditional medicine in different cultures and civilizations [4].

It is used to treat hemorrhoids, herpes zoster, cough, and indigestion [5]. Numerous therapeutic benefits are associated with this plant including antibacterial, antiviral, anticancer, anti-inflammatory, and anti-liver fibrosis activities [6,7].

The medicinal properties of *Persicaria* *Perfoliata* and any other plants are due to bioactive phytochemicals present in the plant. Polyphenols are a group of secondary metabolites with antioxidant properties that work well to reduce oxidative stress. A polyphenolic compound called resveratrol has exhibited a blood glucose-lowering effect on obese men with insulin resistance [8]. In another study, daily oral administration of green tea catechin capsules prevented the formation of tumors in men with high-grade prostate epithelial neoplasia [9]. Similarly, consumption of 60 IU/day vitamin E reduced the risk of coronary heart disease in men by 40 % compared to individuals receiving 7.5 IU/day [10]. Antioxidant properties of medicinal plants protect us from different chronic diseases by preventing DNA damage and low-density lipoprotein (LDL) oxidation [11].

Cancer is a global health problem that results from alcohol and tobacco consumption, diet, radiation, pollution, and lifestyle. Over 3000 plant species are known to have anticancer properties, and over 25 % of prescription medications worldwide are derived from plants [12].

The anticancer activity in medicinal plants involves programmed cell death identified by a specific set of morphological features, such as membrane leaking, nuclear and cytoplasmic shrinkage, and nuclear DNA damage in cells as a result of endonuclease activation [13]. Plant secondary metabolites and green synthesized nanoparticles are also reported for antidiabetic activity [14]. A primary mechanism of antidiabetic activity involves inhibition of digestive enzymes such as α-amylase and α-glucosidase [15].

The word "nanotechnology" refers to the creation, representation, manipulation, and use of structures through the control of shape and size at the nanoscale level [16]. The green synthesis of nanoparticles (NPs) is increasing worldwide, and nanotechnology is the most active and interesting area of material science study. NPs exhibit completely new or improved properties [16]. Gold and silver nanoparticles are becoming increasingly popular because they exhibit excellent properties and practical versatility [17]. They have wide applications in the fields of biology, medicine, water treatment, solar energy conversion, and catalysis.

There are several ways to create silver nanoparticles, such as ion sputtering, sol-gel, and chemical reduction. Unfortunately, these methods require large amounts of toxic chemicals or a lot of energy [18]. Thus, it has become essential to adopt an alternative that is both economical and environmentally friendly. Green synthesis employs sugars, plant extracts, biodegradable polymers, and microbes as capping, stabilizing, and reducing agents to synthesize nanoparticles [19,20]. It has many advantages such as simplicity, a one-step process, cost-effectiveness, eco-friendliness, relative reproducibility, and a tendency to produce more stable materials [21,22]. Green synthesized nanoparticles are reported for their medicinal applications. They can be used as antimicrobial, anticancer, larvicidal, and antioxidant agents [23]. A clinical trial involving 86 men with infected wounds observed complete wound healing in 86 % of participants treated with silver nanoparticle-based cream (KAdermin) compared to 61.5 % of the participants treated with Mupirocin [24]. The shape, size, aggregation, solubility, surface area, and morphology of synthesized Ag NPs can be determined by using different spectroscopic techniques [25]. These include energy dispersive analysis (EDX) of X-rays, atomic force microscopy (AFM), transmission electron microscopy (TEM), scanning electron microscopy (SEM), Fourier transform infrared spectroscopy (FTIR), ultraviolet–visible spectroscopy (UV–vis), and X-ray diffraction (XRD) techniques [26].

The extensive literature review on the biological aspects of *Persicaria perfoliata* discovered a lack of publications on this plant's activities. There are very few reports regarding the antioxidant, antimicrobial, antidiabetic, cytotoxic activity, and plant-mediated synthesis of nanoparticles. Therefore, the present study explores the phytochemical and biological activities in *Persicaria perfoliata* aqueous extract synthesized Ag NPs. The synthesis of silver nanoparticles using this unexplored traditional medicinal plant and exploring antioxidant, antibacterial, toxicity and anticancer properties are the novel studies in this research.

## 2 Materials and methods

### 2.1 Glasswares, instruments and chemicals

Glassware like beakers, funnels, test tubes, volumetric flasks, and conical flasks manufactured from type 1 class borosilicate glass from Borosil company were used in this study. Instruments included a hot air oven (Griffin-Grundy), pH meter (NIKE), water bath (Clifton), and a microplate reader (Epoch 2, Biotek, Instruments, Inc., USA). Analytical grade (extra pure) solvents, such as methanol, ethanol, ethyl acetate, dichloromethane, and hexane were procured from Merck and Fischer Scientific. EDTA disodium salt dihydrate was obtained from SRL, boric acid, calcium chloride fused, and sodium chloride was of Merck. Folin Ciocalteu reagent and resazurin (LOBA CHEMI Pvt. Ltd), Mueller Hinton Broth, and Nutrient Agar were of HieMedia.

### 2.2 Plant sample preparation

*Persicaria perfoliata* was collected from Kathmandu, Nepal on June 30th, 2023. The voucher specimen was deposited in the National Herbarium and Plant Laboratories, Godawari, Nepal for identification with a voucher code D-19KATH163148. The Aerial parts of *P. perfoliata* were ground in a rotary mill, and the fine powder was collected into a water-resistant zip-lock bag after the parts were rinsed twice with distilled water, dried in the shade for about two weeks, and then cleaned twice with tap water.

### 2.3 Preparation of plant extract

50 g of finely powdered aerial parts of this medicinal plant was soaked in 500 mL of each of six different solvents (water, methanol, ethanol, dichloromethane, ethyl acetate, and hexane) in a conical flask. The conical flask was shaken every 24 h for 3 days. Then, the contents were filtered and the filtrate was dried using a water bath, maintaining the temperature of 40 °C. The plant extracts were stored at 4 °C in a sealed container for future use. The yield percentage of crude extract was calculated by using the following formula:

$$\%\;\text{Yield}=\frac{\text{Dry}\;\text{weight}\;\text{of}\;\text{extract}}{\text{Dry}\;\text{weight}\;\text{of}\;\text{sample}}\;X\;100$$

### 2.4 Qualitative phytochemical screening

Preliminary screening of phytochemicals was performed by following the standard protocols [27]. The color change was used to detect the presence or absence of a particular group of organic compounds as the plant's secondary metabolites.

### 2.5 Estimation of total phenolic content (TPC)

The total phenolic content of plant extracts was estimated using the Folin-Ciocalteu (FC) phenol reagent method [28]. 20 μL of plant extract, 100 μL of 10 % FC reagent (1:10), and 80 μL of 1 M Na_2_CO_3_ were added to the wells of a 96-well plate. The reaction mixture was allowed to incubate at room temperature for half an hour before a deep blue color was observed. Then, a spectrophotometer was used to measure absorbance at 765 nm. A standard curve was prepared by using different concentrations of gallic acid (7.5–100 μg/mL) and the total phenolic content (TPC) was expressed in milligrams of gallic acid equivalent (mg GAE/g) per gram of dry extract.

### 2.6 Estimation of total flavonoid content (TFC)

The total flavonoid content was estimated by using the aluminum chloride (AlCl_3_) colorimetric method [29]. 20 μL of plant extract was added to 100 μL of distilled water and 60 μL ethanol was taken in the wells of a well plate. It was followed by 10 μL of 10 % AlCl_3_ solution and 10 μL (1M) CH_3_COOK solution. The reaction mixture was incubated for 30 min at room temperature. Then, absorbance was taken at 415 nm with the help of a spectrophotometer. A quercetin (10–100 μg/mL) standard calibration curve was used to calculate TFC and presented as milligrams of quercetin equivalent per gram of the dry extract (mg QE/g).

### 2.7 Determination of antioxidant activity

The antioxidant activity was determined by using the DPPH radical scavenging assay [30]. For this, 100 μL of different concentrations (15.62–500 μg/mL) of the plant extract was added along with the 100 μL of 0.1 mM of DPPH reagent (3.9 mg DPPH in 100 mL). Quercetin was used as a standard compound. The reaction mixture was then allowed to incubate at room temperature for 30 min. Finally, a spectrophotometer was used to measure absorbance at 517 nm. The following formula was used to calculate the % inhibition:

$$\%\;\text{inhibition}=\frac{\text{Absorbance}\;\text{of}\;\text{control}-\text{Absorbance}\;\text{of}\;\text{sample}}{\text{Absorbance}\;\text{of}\;\text{control}}\;X\;100$$

GraphPad Prism software (version 8.0.2.263) was used to calculate 50 % inhibitory concentration (IC_50_).

### 2.8 Determination of antidiabetic activity

The antidiabetic activity was measured by using the DNSA method [15]. Firstly, aqueous extract and Ag NPs were dissolved in a minimum amount of 10 % DMSO to prepare stock solutions. Then, stock solutions were mixed with buffer (pH 6.9) and NaCl to prepare solutions of different concentrations. 200 μL of plant extract solution was mixed with 200 μL of α-amylase and the mixture was incubated at 30 ^○^C for 10 min, followed by adding 200 μL of starch solution (1 % w/v in water). After 3 min of incubation, 200 μL DNSA reagent was added to terminate the reaction, and the mixture was heated at 85–90 ^○^C for 10 min in a water bath. After cooling, the solution was diluted with 5 mL of distilled water. Finally, a UV–visible spectrophotometer was used to take absorbance readings at 540 nm. Blank reading was taken by replacing the volume of plant extract with buffer solution. The percentage inhibition was calculated as:

$$\%\;\text{inhibition}=\frac{\text{Absorbance}\;\text{of}\;\text{control}-\text{Absorbance}\;\text{of}\;\text{sample}}{\text{Absorbance}\;\text{of}\;\text{control}}\;X\;100$$

GraphPad prism software (version 8.0.2.263) was used to calculate 50 % inhibitory concentration (IC_50_).

### 2.9 Determination of antimicrobial activity

The agar well diffusion method was applied to measure antibacterial activity [31]. Mueller Hinton Broth (MHB) was used for the growth of test microorganisms (ATCC 25931 *Shigella sonnei*, ATCC 43300 *Staphylococcus aureus*, ATCC 700603 *Klebsiella pneumonae* and ATCC 25312 *Escherichia coli*). The broth media was incubated at 37 °C for 24 h. After this, Muller Hinton Agar (MHA) plates were inoculated with test organisms (0.5 McFarland standard). Then, 50 μL of plant extract solutions (50 mg/mL in 50 % DMSO) were added to the bores (6 mm) in MHA plates. The plates were then incubated for 18–24 h at 37 °C. After incubation, the zone of inhibition was measured with the help of a scale. Neomycin and 50 % DMSO were used as positive and negative controls respectively.

### 2.10 Determination of minimum inhibitory concentration (MIC) and minimum bactericidal concentration (MBC)

MIC and MBC were determined by using the resazurin microtiter assay [32]. Plant extracts were serially diluted directly in a sterile 96-well microdilution plate with flat bottom wells containing MHB to obtain varying concentrations. The 0.5 McFarland turbidity culture in MHB was diluted 1:100 to give the bacterial inoculum a final concentration of 106 CFU/mL. Lastly, 5 μL of bacteria were injected into every well of 96 well plates. The plate was covered with a sterile lid and incubated for 20–24 h at 37 °C. Then, 0.003 % resazurin dye was added to each well and the mixture was incubated for 3–4 h at 37 °C. The color of the wells with bacterial growth changed to pink, whereas the wells without bacterial growth stayed blue. The MIC was found by locating the lowest concentration at which bacterial growth is inhibited. Finally, MBC was determined by streaking the contents of the wells onto nutrient agar plates and incubating them at 37 °C for 18 h.

### 2.11 Brine shrimp lethality assay (BSLA)

A preliminary assessment of cytotoxicity was performed by using the brine shrimp lethality assay [33]. Brine shrimp eggs were hatched by putting 50 mg of egg in a beaker containing artificial seawater. The beaker was illuminated with a 100-W table lamp and the mixture was left for 48 h at a temperature of 30 °C. Then, 10 healthy larvae were added to test tubes containing different concentrations of plant extracts and Ag NPs in artificial seawater. The number of surviving nauplii was counted after 24 h and the concentration lethal to 50 % of test organisms (LC_50_) was calculated by linear regression analysis of percentage mortality versus concentration curve using Origin 2024b software.

### 2.12 MTT assay

The cytotoxic activities of the Ag NPs were assessed using the standard MTT (3- [4, 5-dimethylthiazole-2-yl]-2, 5-diphenyl-tetrazolium bromide) reagent with minor modifications [34]. The assay was performed against cervical cancer (HeLa) and lung cancer (A549) cell lines. The cells were grown in DMEM in T flasks with a 5 % CO₂ incubator at 37 °C supplemented with 10 % fetal bovine serum (FBS), 1 % penicillin/streptomycin as an antibiotic, and 1 % L-glutamine. The cells were loaded in 96-well plates (1x104 cells/well) with 100 μL of medium and incubated in a 5 % CO_2_ incubator at 37 °C for 24 h. Then, the cells were treated with varying doses of the Ag NPs solutions (12.5, 25, 50, and 100 μg/mL) for 48 h after attachment and cell confluence. After the incubation period of 48 h, the supernatant was extracted and 100 μL of media containing 20 μL of MTT was introduced into each well. After incubation for 4 h, a purple formazan product was generated. To dissolve formazan, 100 μL of DMSO (0.1 %) was added, and it was then incubated for a further 15 min at room temperature. The absorbance was measured at 570 nm with an (Azure Biosystems Microplate Spectrophotometer) microplate reader. The percentage cytotoxicity and cell proliferation were calculated as:

$$\%\;\text{Cytotoxicity}=\frac{\text{Absorbance}\;\text{of}\;\text{control}-\text{Absorbance}\;\text{of}\;\text{sample}}{\text{Absorbance}\;\text{of}\;\text{control}}\;X\;100$$

Half maximal inhibitory concentration (IC_50_) was calculated by linear regression analysis of the dose-response curve using Origin 2024b software.

### 2.13 Preparation of aqueous plant extract

The powder (5 g) of the *P. perfoliata* was dissolved in 100 mL of distilled water taken in an Erlenmeyer's flask. The mixture was heated for 20 min using a magnetic hot stirrer. An aqueous extract was obtained by filtering the content through Whatman no. 1 filter paper.

### 2.14 Plant-assisted synthesis of silver nanoparticles

10 mL of an aqueous extract was added to 90 mL of AgNO_3_ solution (1 mM). Then, the solution was heated on a heating plate with a magnetic stirrer for 20 min. The final pH of the solution was maintained at 10 and the mixture was continuously stirred at room temperature for almost half an hour. The reaction mixture was changed from light yellow or brown to strong brown indicating the formation of silver nanoparticles in the solution. A UV–vis spectrophotometer indicated the preliminary formation of silver nanoparticles showing an absorption peak within 300–600 nm, measuring the surface plasmon resonance (SPR).

### 2.15 Characterization of Ag NPs

#### 2.15.1 UV visible spectroscopy

The absorption spectra (300–600 nm) of synthesized Ag NPs were analyzed with UV–visible spectroscopy using a UV–vis spectrophotometer (SPECORD 200 PLUS, an Endress + Hauser Company), and Origin Pro 2024 was utilized to plot the absorption results.

#### 2.15.2 Fourier transform infrared spectroscopy (FTIR)

The functional groups of organic compounds in the plant extract as secondary metabolites were analyzed by using the FTIR as the characterization technique having a scanning range between 400 and 4000 cm^−1^ (Nicolet iS50 FTIR) and the data was analyzed using the software Origin Pro 2024.

#### 2.15.3 X-ray diffraction (XRD)

The degree of crystallinity, crystallite size, and diffraction pattern of synthesized silver nanoparticles were characterized by the X-ray diffraction (XRD) technique (D2phaser Bruker, NAST, Nepal) having wavelength 1.540 A° and 30 KV diffractometer. Within the (20–80) degree range scanning 2 theta was carried out. The crystallite size of synthesized silver nanoparticles was determined by using Scherrer's equation as follows:

$$D=\frac{K\lambda }{\beta \;\mathrm{Cos}\;\theta }$$

Where,

D = Crystallite size in nm.

K = dimensionless shape factor, with a value close to unity (0.9)

*λ* = Wavelength of X-ray radiation used, (0.15406 nm for Cu Kα).

Θ = Bragg's angle (half of 2θ value of chosen peak; in radians).

Β = Full-width half maximum (FWHM; in radians)

#### 2.15.4 Field emission scanning electron microscopy (FE-SEM)

Surface topography, grain size, and other physical characteristics of nanoparticles were determined by using the FE-SEM images. The grain size of synthesized silver nanoparticles was determined with the help of Image J software.

#### 2.15.5 Energy dispersive X-ray analysis (EDX)

An energy-dispersive X-ray analysis method was used to determine the elemental composition of the synthesized silver nanoparticles. For this reason, the presence of silver nanoparticles is critical for both the FE-SEM and EDX analysis.

## 3 Results and discussion

### 3.1 Percentage yield

The percentage yield of plant extracts in different solvents is shown in Table 1. An aqueous extract had the highest percentage yield at 14.09 % followed by 11.18 % for methanol extract, 5.76 % for ethanol extract, 3.51 % for ethyl acetate, 2.53 % for DCM, and 0.84 % for hexane extract.

**Table 1:** The percentage yield of the extracts of aerial parts of *Persicaria perfoliata*.

Extracts	Percentage yield
Aqueous extract	14.09
Methanol extract	11.18
Ethanol extract	5.76
Ethyl acetate extract	3.51
DCM extract	2.53
Hexane extract	0.84

### 3.2 Qualitative phytochemical analysis

The results of the qualitative phytochemical screening of an aqueous extract are presented in Table 2. Preliminary screening revealed the presence of alkaloids, carbohydrates, reducing sugar, glycosides, amino acids, flavonoids, phenols, tannins, terpenoids, anthraquinones, and phytosterols in the aqueous extract of *Persicaria perfoliata*. These phytochemicals are known to possess various medicinal properties. Studies have also identified their role as capping, reducing, and stabilizing Ag ions during nanoparticle synthesis [19,20]. The presence of these phytochemicals is responsible for biological activities and Ag NPs synthesizing capacity of the organic compounds present in the extracts of *Persicaria perfoliata*.

**Table 2:** Preliminary qualitative phytochemical analysis.

Phytochemicals	Crude extracts
Alkaloids	+
Carbohydrates	+
Reducing sugars	+
Glycosides	+
Amino acids	+
Flavonoids	+
Phenols	+
Tannins	+
Terpenoids	+
Anthraquinones	+
Phytosterols	+

+ indicates the presence of phytochemicals.

### 3.3 Total phenolic content (TPC) and total flavonoid content (TFC)

The TPC and TFC in various solvent extracts of *Persicaria perfoliata* were determined, as presented in Table 3. The methanol extract exhibited the highest TPC, with a value of 68.61 ± 0.57 mg GAE/g, whereas the hexane extract showed the lowest TPC of 26.35 ± 2.76 mg GAE/g. Similarly, the TFC was highest in the methanol extract, recorded as 40.69 ± 5.0 mg QE/g, while the dichloromethane (DCM) extract demonstrated the lowest TFC, measuring 5.54 ± 2.27 mg QE/g. These findings suggest that the significant levels of phenolics and flavonoids in the different solvent extracts contribute to the pharmacological potential of *P. perfoliata.*

**Table 3:** TPC and TFC in different solvent extracts of *P. perfoliata*.

Extracts	Total phenolic content (mg GAE/g)	Total flavonoid content (mg QE/g)
Aqueous extract	52.17 ± 3.66^a,b^	17.51 ± 3.02^a,b^
Methanol extract	68.61 ± 0.57^c^	40.69 ± 5.0
Ethanol extract	60.04 ± 6.62^b,c^	22.93 ± 6.3^a^
Ethyl acetate extract	43.36 ± 3.71^a^	7.81 ± 1.98^b,c^
DCM extract	31.07 ± 5.85^d^	5.54 ± 2.27^c^
Hexane extract	26.35 ± 2.76^d^	11.6 ± 3.28^b,c^

Values followed by different letters or no letters are significantly different from each other at p < 0.05.

### 3.4 Antioxidant activity

**Fig. 1:**
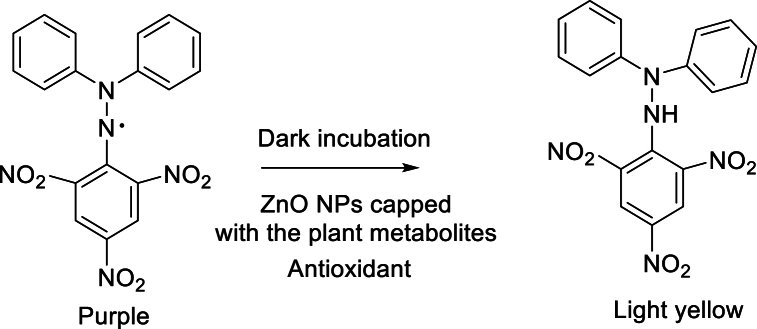
Showing the mechanism of DPPH activity.

**Fig. 2:**
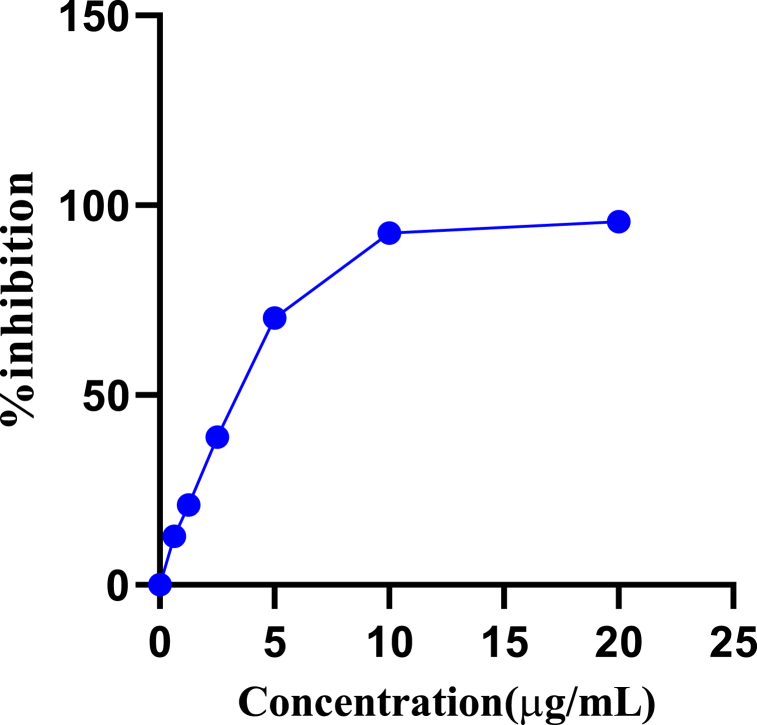
Standard curve of DPPH inhibition by quercetin.

**Fig. 3:**
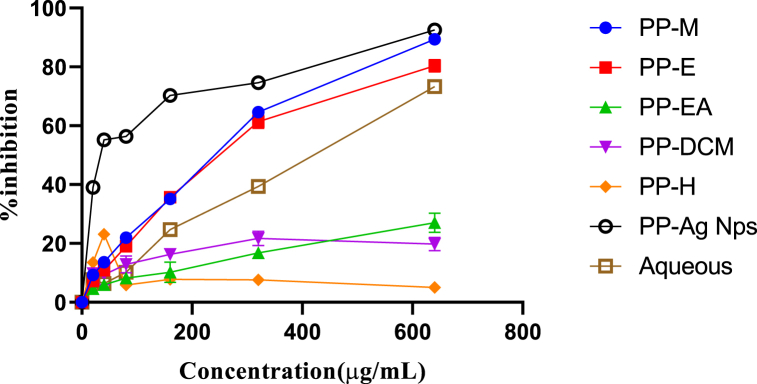
Percentage inhibition versus concentration curve for plant extracts and Ag NPs.

Antioxidant activity in plant extract was determined by DPPH assay. The assay involves a color change of the DPPH solution from violet to light yellow Fig. 1. It is caused by the transfer of a single hydrogen atom from an antioxidant compound to a DPPH free radical to form a stable compound. The reaction involved in the DPPH assay is given as:

The antioxidant potential as the IC_50_ values for different solvent extracts, Ag NPs, and standard quercetin are provided in Table 4. The lowest IC_50_ value at 69.40 ± 0.13 μg/mL was recorded for hexane extract followed by DCM extract with 116.5 ± 1.63 μg/mL, whereas ethyl acetate extract had the highest IC_50_ of 3036 μg/mL. The IC_50_ values of the standard quercetin and Ag NPs were 3.43 ± 1.61 μg/mL and 144.5 ± 1.36 μg/mL. The increasing order of antioxidant activity can be presented as quercetin < hexane < dichloromethane < ethanol < AgNPs < methanol < aqueous < ethyl acetate. Fig. 2, Fig. 3 present concentration-dependent increments in percentage radical inhibition with concentrations of standard and plant extracts.

**Table 4:** IC_50_ values for plant extracts and nanoparticles against DPPH and α-amylase inhibition.

Extracts	IC_50_ (μg/mL)	
	Antioxidant activity	α-amylase inhibition
Aqueous extract	452.5 ± 2.82	1188.83 ± 33.52
Methanol extract	185.5 ± 0.99	–
Ethanol extract	123.1 ± 1.03	–
Ethyl acetate extract	3036 ± 0.00	–
DCM extract	116.5 ± 1.63	–
Hexane extract	69.40 ± 0.13	–
Ag NPs	144.5 ± 1.36	1369.30 ± 46.86
aQuercetin	3.43 ± 1.61	–

a Positive control. – Values not measured. Values are significantly different from each other at p < 0.05.

In addition to their direct impact of reducing oxidative damage, antioxidants can also have an indirect effect by increasing the expression or activity of intracellular antioxidant enzymes or lowering that of enzymes that produce free radicals. Intracellular antioxidant enzymes are a crucial defense against reactive oxygen species. These enzymes, which the cell produces, are a vital line of defense against free radicals. SOD, CAT, GPX, glutathione reductase (GRD), glutathione S-transferase (GST), thioredoxin reductase (TrxR), heme oxygenase, and biliverdin reductase are among the most important antioxidant enzymes. Antioxidant compounds protect us from chronic diseases such as cancer and neurodegenerative disorders [35].

### 3.5 Correlation of antioxidant potential with polyphenols and flavonoids

**Fig. 4:**
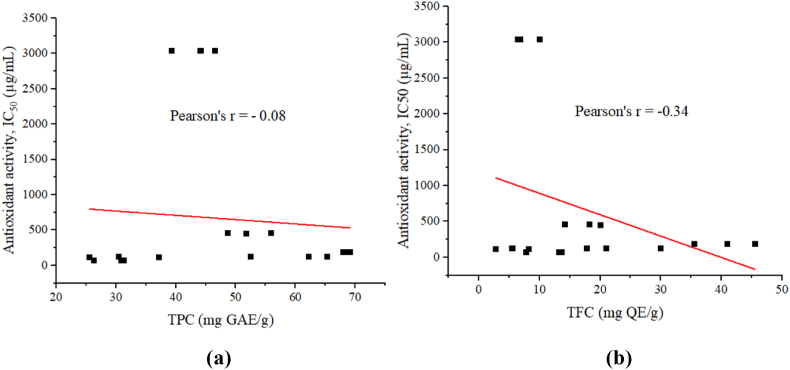
(a) Correlation between TPC and antioxidant activity, and **(b)** correlation between TFC and antioxidant activity.

Phenolic and flavonoid compounds are known to contain antioxidant properties. They can donate a single electron or hydrogen through their hydroxyl group to stabilize reactive oxygen species [11]. In the present study, weak correlations (Pearson's r = −0.08 and −0.34) were observed between IC_50_ values and TPC, and TFC. The prevalence of other antioxidant compounds such as vitamins and co-enzymes may be responsible for such weak correlations. The correlation diagrams are presented in Fig. 4 (a) and **(b)**

### 3.6 Antidiabetic activity

**Fig. 5:**
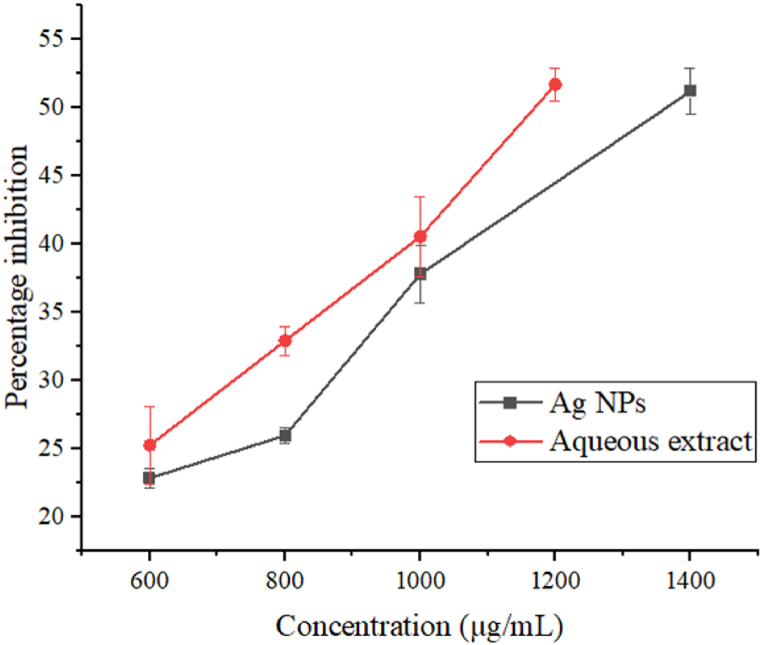
Percentage inhibition versus concentration in α-amylase assay.

The ability of Ag NPs and aqueous extract to inhibit α-amylase enzyme was measured to assess their antidiabetic activity. The α-amylase inhibitory activity as in IC_50_ values is provided in Table 4. Higher IC_50_ value for Ag NPs (1369.30 ± 46.86 μg/mL) and aqueous extract (1188.83 ± 33.52 μg/mL) indicates a weaker α-amylase inhibitory activity. In contrast, *Annona muricata* synthesized Ag NPs displayed strong antidiabetic activity with an IC_50_ value of 26.31 ± 3.99 μg/mL [36]. Another study on Ag NPs synthesized using *Tephrosia tinctoria* also reported significant antidiabetic activity [14]. These differences in activities may have resulted from the physiochemical characteristics of synthesized nanoparticles and the medicinal plant used in the study. Aqueous extract displayed comparatively higher antidiabetic activity than Ag NPs. The presence of some antidiabetic secondary metabolites in aqueous extract may have caused higher activity. Concentration-dependent increments in the percentage of α-amylase inhibition by aqueous extract and Ag NPs are provided in Fig. 5.

### 3.7 Evaluation of antimicrobial activity

**Fig. 6:**
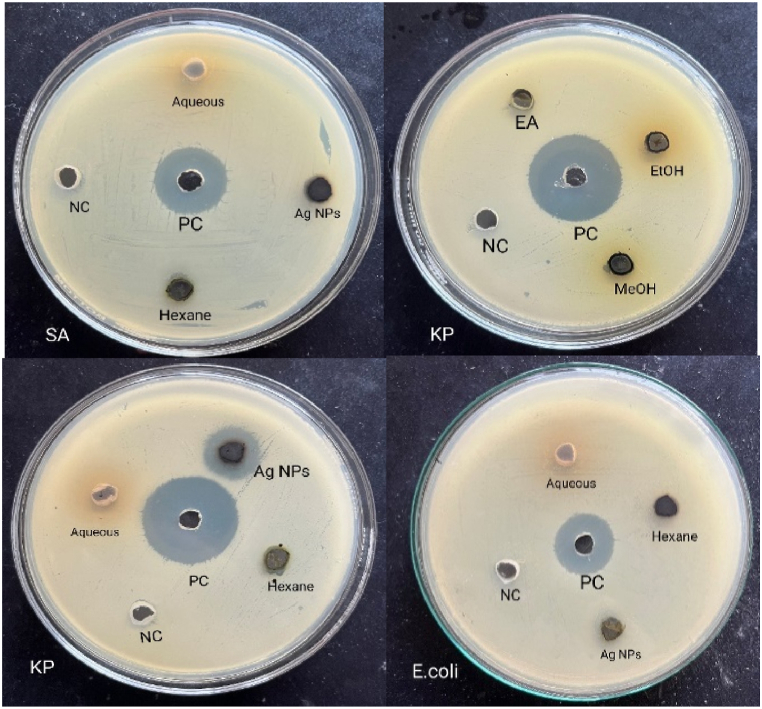
ZOI shown by the plant extracts and silver nanoparticles against test organisms.

The zone of inhibition of different crude extracts and nanoparticles against four different bacteria is illustrated in Table 5 and Fig. 6. The synthesized Ag NPs were effective against *K. pneumoniae* with ZOI 16 mm. ZOI of 11 mm was displayed by aqueous extract against *K. pneumoniae* and methanol extract against *S. sonnei*. ZOI of 9 mm was recorded for all extracts against *S. aureus* and *E. coli*. The antibacterial action of medicinal plant materials is probably due to the presence of alkaloids and polyphenols.

**Table 5:** ZOI showed by various extracts of *P. perfoliata* and nanoparticles against test organisms.

Solvent extract	Zone of inhibition (mm)			
	*K. pneumoniae*	S. aureus	*E. coli*	*S. sonnei*
Aqueous extract	11	9	9	9
Methanol extract	9	9	9	11
Ethanol extract	9	9	9	10
Ethyl acetate extract	9	9	9	9
Hexane extract	9	9	9	9
Ag NPs	16	9	9	9
aNeomycin	25	20	23	20

a Positive control.

### 3.8 MIC and MBC

**Fig. 7:**
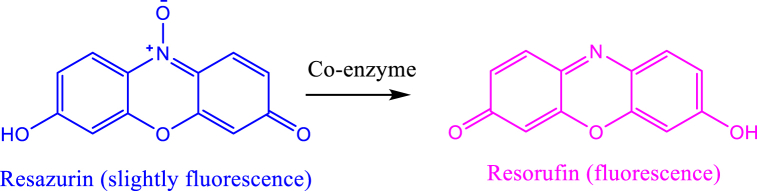
Showing the colour change in a resazurin microtiter assay. (For interpretation of the references to color in this figure legend, the reader is referred to the Web version of this article.)

**Fig. 8:**
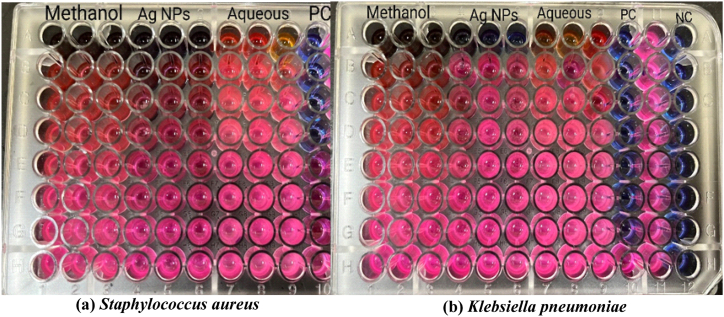
MIC of methanol extract, aqueous extract, and Ag NPs against **(a)***Staphylococcus aureus* and **(b)***Klebsiella pnemoniae* shown in 96 well plates.

**Fig. 9:**
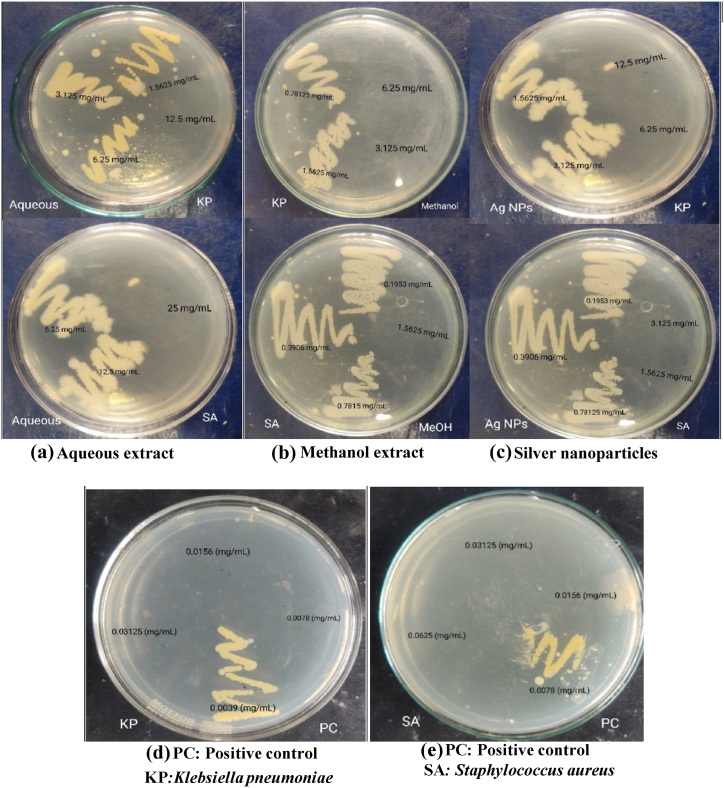
Determination of MBC of an **(a)** aqueous extract, **(b)** methanol extract and **(c)** Ag NPs **(d), and (e)** positive control against KP: *Klebsiella pneumoniae* and SA: *Staphylococcus aureus*.

The MIC and MBC of methanol extract, aqueous extract, and silver nanoparticles were determined by using the resazurin microtiter assay. The assay involved the reduction of resazurin dye into resorufin by the mitochondrial NADH enzyme. This reduction is accompanied by a color change from purple to pink Fig. 7. The reaction involved in this process is given as:

The gram-positive bacteria *Staphylococcus aureus* and gram-negative *Klebsiella pneumoniae* were used in the study. Against *Klebsiella pneumoniae*, the MIC of methanol extract, aqueous extract, and silver nanoparticles were determined to be 1.5625, 6.25, and 3.125 mg/mL, respectively Table 6. The MIC of methanol, aqueous, and silver nanoparticles against *Staphylococcus aureus* were found to be 0.78125, 12.5, and 0.78125 mg/mL respectively. The MBC values of methanol extract, aqueous extract, and synthesized silver nanoparticles were 3.125, 12.5, and 6.25 mg/mL against *Klebsiella pneumoniae* and 1.5625, 25, and 1.5625 mg/mL against *Staphylococcus aureus*. The positive control neomycin displayed MIC and MBC of 0.0039 mg/mL and 0.0078 mg/mL against *Klebsiella pneumoniae* and 0.0078 mg/mL and 0.0156 mg/mL against *Staphylococcus aureus*. Ag NPs displayed enhanced MIC and MBC compared to an aqueous extract. The photographs of microtiter plates and MHA plates are provided in Fig. 8, Fig. 9.

**Table 6:** MIC and MBC of plant extracts and Ag NPs.

Extracts	*Klebsiella pneumoniae*		*Staphylococcus aureus*	
	MIC (mg/mL)	MBC (mg/mL)	MIC (mg/mL)	MBC (mg/mL)
Methanol extract	1.5625	3.125	0.78125	1.5625
Ag NPs	3.125	6.25	0.78125	1.5625
Aqueous extract	6.25	12.5	12.5	25
aNeomycin	0.0039	0.0078	0.0078	0.0156

a Positive control.

### 3.9 Mechanism of Ag NPs against the bacterial strains

**Fig. 10:**
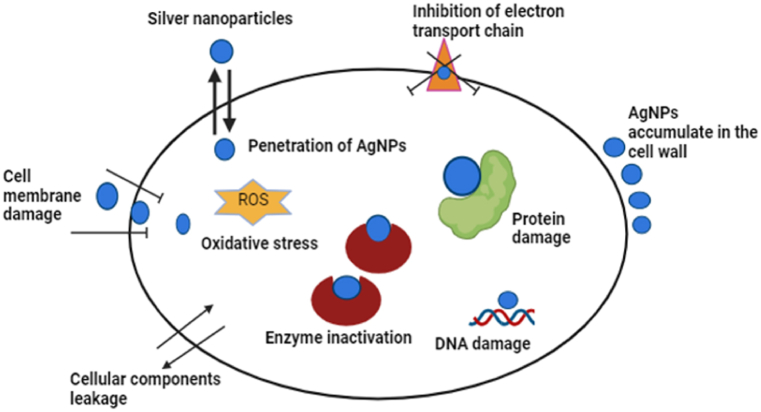
Diagrammatic representation of the role of silver nanoparticles in the generation of ROS leading destruction of bacterial cells.

One of three models is commonly used to explain the antibacterial mechanism of action of NPs. They are oxidative stress induction, metal ion release, or non-oxidative processes. All three of these mechanisms can operate at once. According to some research, Ag NPs cause the bacterial membrane's surface electric charge to be neutralized, altering the penetrability of the cell membrane and eventually causing bacterial mortality [37]. Furthermore, the production of reactive oxygen species (ROS) damages the cell membrane mechanically and suppresses the antioxidant defense system. The main mechanisms underlying the antimicrobial effects of NPs involve disruption of the bacterial cell membrane, production of reactive oxygen species (ROS), penetration of the bacterial cell membrane, and induction of intracellular antibacterial effects, such as interactions with DNA and proteins. Some of these bacterial death mechanisms by the NPs include membrane contact, ATP depletion, biomolecule destruction, reactive oxygen species formation, and cation release [37]. A diagrammatic representation of the antibacterial mechanism of Ag NPs is provided in Fig. 10.

### 3.10 Plant-mediated synthesis of silver nanoparticles and characterization

The shifting of the color of the solution indicates the formation of silver nanoparticles. The formation of silver nanoparticles was confirmed by using the following characterizing techniques.

#### 3.10.1 UV visible spectroscopy

**Fig. 11:**
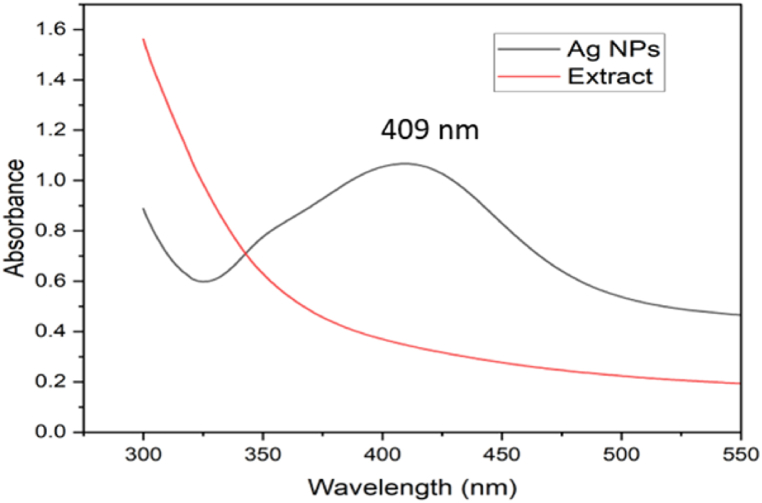
UV visible spectra of synthesized silver nanoparticles and aqueous extract.

Depending on their size, shape, and morphology, Ag NPs display a strong surface plasmon resonance (SPR) in an aqueous solution and absorb within 300–600 nm [38]. Ag NPs synthesized in the present study displayed a visible absorption maximum at 409 nm Fig. 11. Such absorption maxima were absent in the spectra of aqueous extract. Thus, the spectra confirm the formation of Ag NPs. A previous study on the Ag NPs synthesized using *Rubus ellipticus* extract had reported an absorption peak in the range of 416–420 nm whereas the SPR peak for the Ag NPs prepared using *Annona muricata* was reported at 420 nm [39,40]. Small shifts in the absorption spectra of Ag NPs may occur due to variations in temperature, pH, extract concentration, and light intensity levels [41].

#### 3.10.2 Fourier transform infrared spectroscopy (FTIR)

**Fig. 12:**
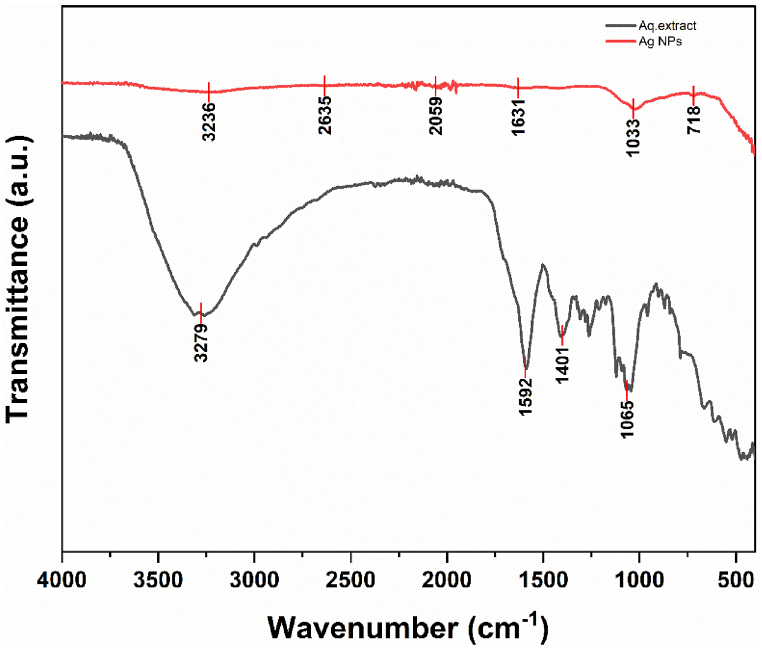
FTIR spectra of plant extract and silver nanoparticles.

The FTIR helps with the identification of functional groups, H-bonding, and metal-oxygen bond formation. Comparative FTIR spectra between plant extract and silver nanoparticles play a crucial role in the verification of the presence of silver nanoparticles and the involvement of functional groups. The diverse range of functional groups present in the plant extract and synthesized nanoparticles were identified by FTIR and these functional groups act as stabilizing, capping, and reducing agents respectively. Aqueous extract displayed peaks at 3277 cm^−1^, 1590 cm^−1^, 1401 cm^−1^, and 1065 cm^−1^ Fig. 12. Whereas Ag NPs contained peaks at 3235 cm^−1^, 2632 cm^−1^, 2059 cm^−1^, 1631 cm^−1^ and 1024 cm^−1^. In plant extract, the peak at 3277 cm^−1^ revealed the presence of a strong –OH bond, the peak at 1590 cm^−1^ revealed –C=C- stretching, and the peak at 1065 cm^−1^ shows there was the occurrence of C-O stretching. The FTIR spectra of synthesized nanoparticles are similar to the aqueous plant extract with slight modification that indicates the involvement of functional groups in nanoparticle synthesis. The peak at 3235 cm^−1^ represents the presence of the –OH functional group which is a broad beak but the –OH group is also present in plant extract but there is a narrow-pointed peak. The peak at 2632 cm^−1^ represents the –C-H stretching and the peak at 1631 cm^−1^ represents –C=C- stretching. Extracts include flavonoids, phenolic, and other bioactive substances that are involved in the synthesis of Ag NPs [42]. *Annona muricata* synthesized Ag NPs displayed FTIR peaks at 610.77, 1075.83, 1384.58, 1640.17, 2921.03, and 3381.28 cm^−1^ [43].

#### 3.10.3 X-ray diffraction (XRD)

**Fig. 13:**
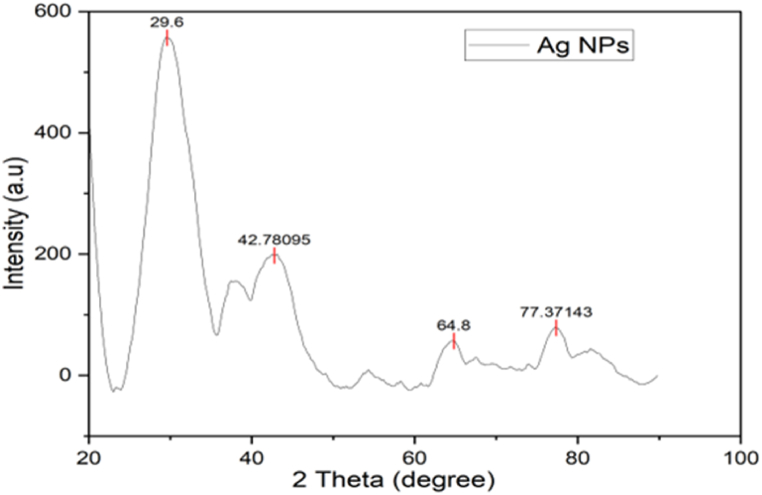
XRD pattern of plant-assisted synthesized silver nanoparticles.

The crystal structure and crystallite size of nanoparticles were determined with XRD analysis. One intense and three noticeable peaks have appeared at the 2θ angles of 29.6, 42.78095, 64.8, and 77.37143 respectively in the XRD plot of Ag NPs provided in Fig. 13. The crystallite size of synthesized silver nanoparticles was calculated as 1.54 nm using Debye Scherrer's equation.

#### 3.10.4 Field emission scanning electron microscopy (FE-SEM)

**Fig. 14:**
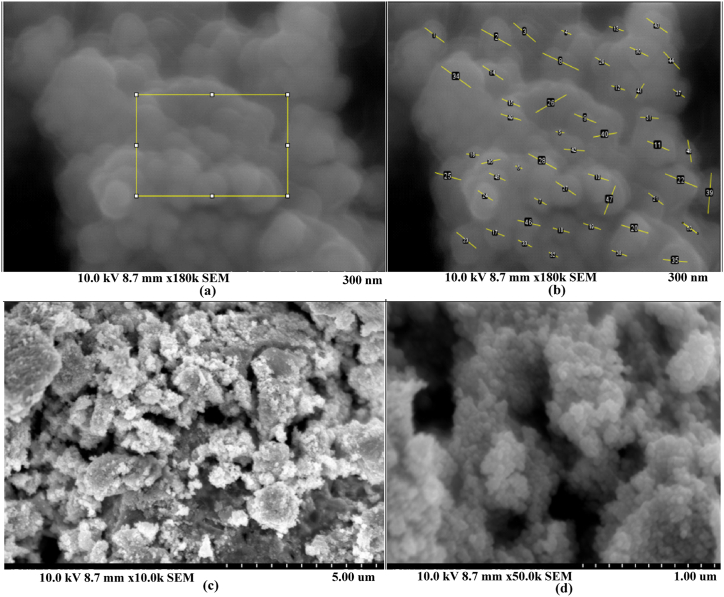
FE-SEM images of the synthesized silver nanoparticles using *P. perfoliata***(a)** 300 nm scale **(b)** measuring the length of Ag NPs **(c)** 5.00 μm scale, and **(d)** 1.00 μm scale.

**Fig. 15:**
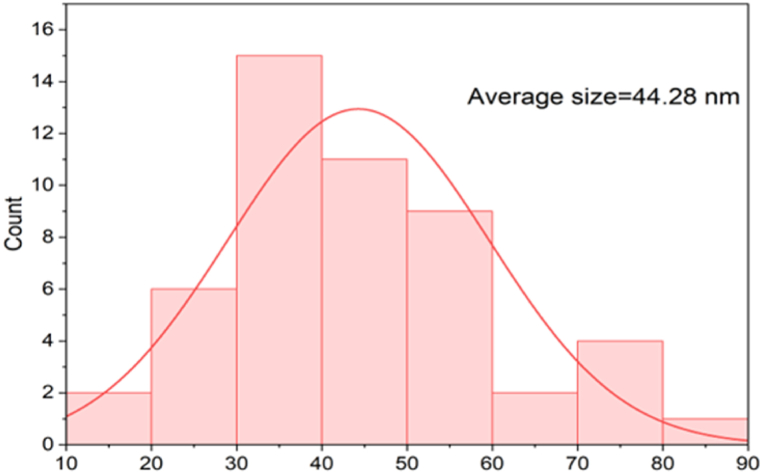
Size distribution of silver nanoparticles.

The grain size and surface morphology of the synthesized silver nanoparticles were determined using the FESEM images. The synthesized nanoparticles displayed irregular morphology and quasi-spherical shape with agglomeration. The average grain size of silver nanoparticles was 44.28 nm. Previously, the grain size for the *Annona muricata* synthesized Ag NPs was reported in the range of 20–30 nm whereas *Rubus ellipticus* synthesized Ag NPs ranged from 13.85 to 34.30 nm [36]. The shape, size, and concentration of synthesized nanoparticles are known to affect the properties of nanoparticles. SEM images were produced due to hydrogen bonding and electrostatic interaction between the bioinorganic capping molecules attached to silver nanoparticles. The FE-SEM images of synthesized nanoparticles are illustrated in Fig. 14(a–d), and the histogram showing particle size distribution is presented in Fig. 15.

#### 3.10.5 Energy dispersive X-ray analysis (EDX)

**Fig. 16:**
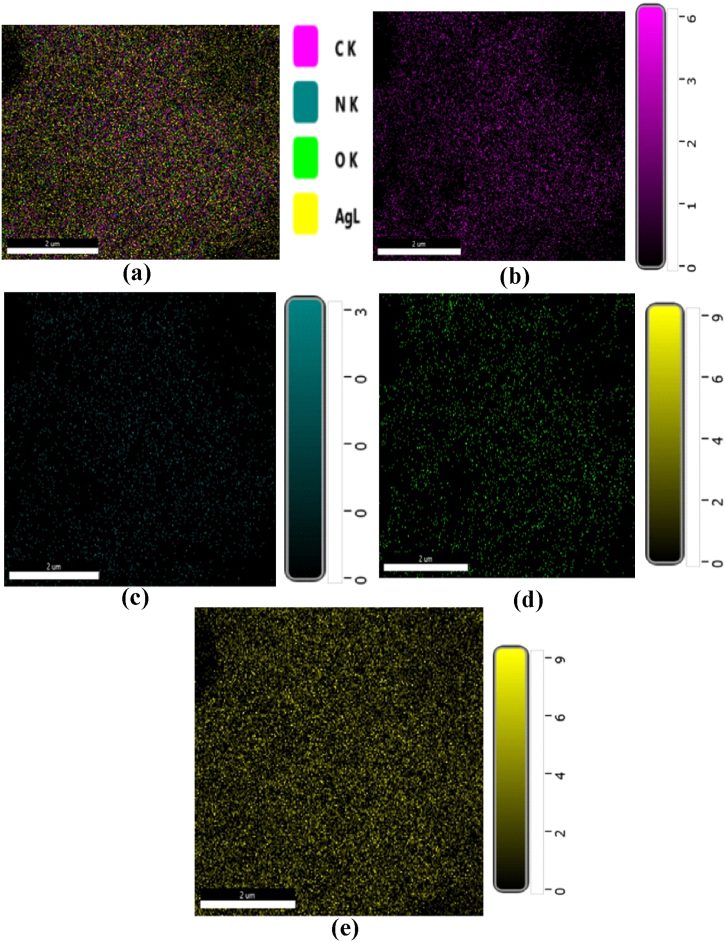
EDX micrographic images **(a)** indicating elemental color mapping of carbon, nitrogen, oxygen, and silver elements **(b)** indicating elemental color mapping of carbon element **(c)** indicating elemental color mapping of nitrogen element **(d)** indicating elemental color mapping of oxygen element **(e)** indicating elemental color mapping of the silver element with the region of interest on the side. (For interpretation of the references to color in this figure legend, the reader is referred to the Web version of this article.)

**Fig. 17:**
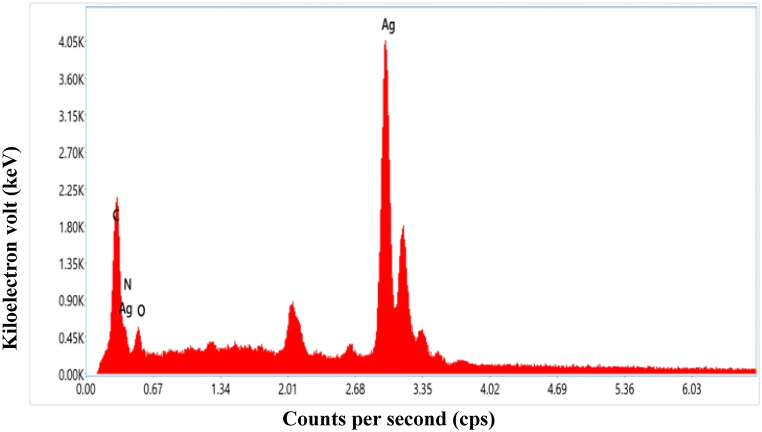
EDX spectrum of synthesized silver nanoparticle using *P. perfoliata*.

EDX analysis was used to determine the chemical composition of synthesized silver nanoparticles Fig. 16(a–e), and Fig. 17 represent the elemental mapping and EDX spectrum. From the EDX spectrum**,** the peaks of silver and oxygen were identified and their % weights were 84.2 % and 2.9 % respectively. The % weight of carbon and nitrogen were recorded as 112 % and 1.7 % respectively Table 7. The existence of silver nanoparticles was shown by a prominent absorption peak at 3 Kev. The detection of carbon, nitrogen, and oxygen in the EDX spectrum of the nanoparticles indicates the presence of secondary metabolites on their surface. Previous studies have also found the presence of these elements in synthesized Ag NPs [44,45].

**Table 7:** Composition of different elements in the synthesized Ag NPs.

Element	Weight %	Atomic %
C K	112	46.3
N K	1.7	5.9
O K	2.9	9.1
Ag L	84.2	38.8

### 3.11 Toxicity

**Fig. 18:**
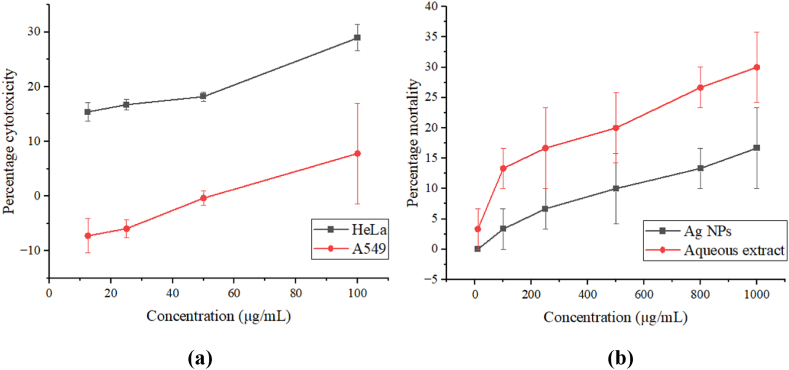
(a) Percentage cytotoxicity versus concentration for MTT assay, and **(b)** Percentage mortality versus concentration graph for brine shrimp assay.

The preliminary assessment of cytotoxicity was performed by measuring the ability of plant extract and Ag NPs to kill brine shrimp nauplii. The LC_50_ values for aqueous extract and Ag NPs were recorded as 1873.54 ± 543.10 and 3486.55 ± 1378.94 μg/mL respectively Table 8. Observed IC_50_ values indicate a weak brine shrimp lethality in both samples. Comparatively lower LC_50_ value in aqueous extract may have resulted from the presence of phytochemicals such as alkaloids. Fig. 18 (a) and **(b)** represent concentration-dependent increments in the percentage mortality shown by the Ag NPs and an aqueous extract against the cancer cell lines HeLa and A549, and the brine shrimp lethality respectively.

**Table 8:** LC_50_ of silver nanoparticles and aqueous extract of *P. perfoliate*.

Samples	LC_50_ (μg/mL)
Ag NPs	3486.55 ± 1378.94
Aqueous extract	1873.54 ± 543.10

Values are not significantly different from each other at p > 0.05.

### 3.12 Anticancer activity

The cytotoxicity of Ag NPs against HeLa and A549 cell lines was tested using the MTT assay. Ag NPs displayed weak cytotoxicity against both cell lines, a fact foreshadowed by the brine shrimp lethality assay. The nanoparticles displayed IC_50_ values of 251.86 ± 58.90 and 1350.12 ± 1644.09 μg/mL against HeLa and A549 cell lines respectively Table 9. *Annona muricata* synthesized Ag NPs displayed an IC_50_ value of 36.53 μg/mL against HeLa cell lines [46]. Nanoparticles synthesized using traditional anticancer medicinal plants have shown remarkable activities against different types of cancer cells [47]. These nanoparticles are generally effective in targeting cancer cells and they show minimal side effects [47]. The cytotoxicity of Ag NPs is influenced by particle size, concentration, and agglomeration of nanoparticles [48]. These *in-vitro* studies should be correlated with *in-vivo* studies to advance the drug discovery process and improve product quality [49].

**Table 9:** IC_50_ values of silver nanoparticles against HeLa and A549 cell lines.

Cell lines	IC_50_ (μg/mL)
HeLa	251.86 ± 58.90
A549	1350.12 ± 1644.09

Values are not significantly different from each other at p > 0.05.

#### 3.12.1 Mechanism of anticancer activity shown by Ag NPs

**Fig. 19:**
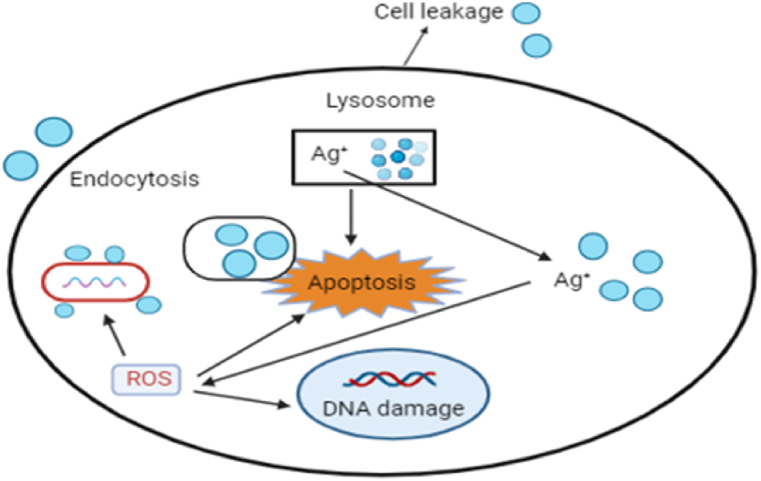
Mechanism of anticancer activity shown by Ag NPs.

The generation of ROS in the cells by Ag NPs is responsible for their anticancer properties and triggers apoptosis, autophagy, and necrosis [50]. Due to their greater surface area, they release silver ions more quickly and trigger ROS and apoptosis. The small Ag NPs are more toxic. The high surface area of nanoparticles enables improved metal action and penetration [51]. The release of silver ions from nanoparticles and the effects of surface charge are combined to produce the cytotoxicity mechanism of Ag NPs. The surface charge of Ag NPs plays a crucial role in how cells build their responses to stress. It is important for binding proteins, which modifies the chemical and physical characteristics of Ag NPs. The toxicity of nanoparticles increases with an increase in surface charge [52]. The subsequent nuclear fragmentation, results from the generation of ROS. Oxidative stress induces physiological and cellular processes such as cell leakage, the lysosome Ag^+^ ion endocytosis ROS, apoptosis, and DNA damage. Ag NPs' anticancer activity mechanism is shown in Fig. 19 which includes DNA damage, apoptosis, stress, mitochondrial disruption, destruction, and inflammation. ROS levels are implicated in DNA damage and illustrate the activation of apoptosis [53].

## 4 Conclusion

The present study on *Persicaria perfoliata* confirms the plant's rich phytochemical profile, featuring phenols, flavonoids, terpenoids, alkaloids, carbohydrates, reducing sugars, glycosides, amino acids, tannins, anthraquinones, and phytosterols. The solvent extracts demonstrated higher total phenolic content (TPC) and total flavonoid content (TFC), indicating effective extraction of these bioactive compounds. Among the tested samples, the biosynthesized silver nanoparticles (Ag NPs) exhibited superior antioxidant activity compared to methanol, aqueous, and ethyl acetate extracts, highlighting their potential as natural antioxidants. The antidiabetic activity was observed to be weak for both the aqueous extract and Ag NPs. In terms of antimicrobial efficacy, solvent extracts, and Ag NPs displayed activity against both gram-positive and gram-negative bacteria, suggesting their utility as broad-spectrum antibacterial agents. Notably, the Ag NPs and aqueous extract did not exhibit significant toxicity against brine shrimp nauplii, indicating a favorable safety profile in preliminary toxicity assessments. However, the Ag NPs showed cytotoxic effects against HeLa and A549 cell lines, suggesting potential anticancer applications. Characterization of the Ag NPs using UV–visible spectroscopy revealed a surface plasmon resonance (SPR) peak at 409 nm, confirming successful nanoparticle synthesis. FTIR analysis identified functional groups involved in nanoparticle formation, while XRD and FE-SEM confirmed the crystalline structure and grain size. EDX analysis further established the elemental composition of the nanoparticles. Overall, the findings indicate that *P. perfoliata* and its biosynthesized Ag NPs possess promising antioxidant, antimicrobial, and cytotoxic properties, supporting their potential application in medicinal and nanotechnology fields. Future studies focusing on *in vivo* evaluations and mechanistic insights would further validate their therapeutic potential.

## CRediT authorship contribution statement

**Deepak Kumar Shrestha:** Writing – review & editing, Writing – original draft. **Dipak Raj Jaishi:** Writing – review & editing, Data curation. **Indra Ojha:** Writing – review & editing. **Dinesh Raj Ojha:** Writing – review & editing, Data curation. **Ishwor Pathak:** Writing – review & editing, Software, Data curation. **Akash Budha Magar:** Writing – review & editing, Software, Methodology, Formal analysis, Data curation. **Niranjan Parajuli:** Writing – review & editing, Supervision, Methodology, Data curation. **Khaga Raj Sharma:** Writing – original draft, Supervision, Data curation, Conceptualization.

## Additional information

No additional information is available in this paper.

## Data availability statement

All the data generated in this study will be provided on request.

## Funding statement

This research was partly funded by the University Grants Commission (UGC), Nepal with the award no. CRIG-78/79-S and T-01.

## Declaration of competing interest

The authors declare the following financial interests/personal relationships which may be considered as potential competing interests:Khaga Raj Sharma reports financial support was provided by University Grants Commission Nepal. Reports a relationship with that includes:. Has patent pending to. If there are other authors, they declare that they have no known competing financial interests or personal relationships that could have appeared to influence the work reported in this paper.
