# Supplementary material for: Plant assisted synthesis of silver nanoparticles using Persicaria perfoliata (L.) for antioxidant, antibacterial, and anticancer properties
Source: Heliyon. 2024 Nov 21;10(23):e40543. doi: 10.1016/j.heliyon.2024.e40543 (PMC11629186; doi:10.1016/j.heliyon.2024.e40543)
Supplement: Multimedia component 1 [file mmc1.docx]

**Supplementary file**

**Plant-Assisted Synthesis of Silver Nanoparticles Using *Persicaria perfoliata (L.)* for Antioxidant, Antimicrobial, and Anticancer Properties**

# Abstract

*Persicaria perfoliata* (L.) is an herbaceous medicinal plant belonging to the Polygonaceae family. The plant is distributed in Nepal, India, Japan, China, Russia, and Korea. The present study involved the analysis of plant secondary metabolites, synthesis of silver nanoparticles (Ag NPs) using the plant, characterization, and exploration of antioxidant, antidiabetic, antibacterial, and cytotoxic activities. Among six different solvent extracts, the methanol extract displayed the highest total phenolic content (TPC) and total flavonoid content (TFC) of 68.61 ± 0.57 mg GAE/g and 40.69 ± 5.0 mg QE/g respectively. Ag NPs and hexane extract displayed the potential antioxidant activity of IC_50_ 69.40 ± 0.13 and 144.50 ± 1.36 µg/mL in the DPPH assay. The α-amylase inhibition shown by an aqueous extract and the synthesized Ag NPs IC_50_ of 1188.83 ±33.52 and 1369.30 ±46.86 µg/mL respectively. In antibacterial activity, the highest ZOI of 16 mm was displayed by Ag NPs against *Klebsiella pneumoniae* followed by a ZOI of 11 mm for methanol extract against *Shigella sonnei*. Similarly, the lowest MIC and MBC of 0.78125 and 1.5625 mg/mL were recorded for both Ag NPs and methanol extract against *Staphylococcus aureus*. Aqueous extract and Ag NPs did not display significant toxicity against brine shrimp nauplii. Ag NPs displayed an IC_50_ of 251.86 ± 58.90 µg/mL against HeLa cell lines. Biosynthesized Ag NPs showed a distinct peak at 409 nm in UV-visible spectra. FTIR analysis revealed the involvement of different functional groups of the organic compounds present in plant extract as reducing, capping, and stabilizing agents in the synthesis of Ag NPs. XRD analysis confirmed the crystal structure of Ag NPs, whereas the average grain size of 44.28 nm was determined by FE-SEM analysis. EDX spectra established the elemental composition of Ag NPs. The present study shows the synthesized Ag NPs using plant extract impart the potential biological activities as compared to that of the crude extract.

**Keywords:** Antidiabetic, antimicrobial, antioxidant, cytotoxicity, green synthesis, *Persicaria perfoliata*, silver nanoparticles

**1. Introduction**

**Table 1**

The percentage yield of the extracts of aerial parts of *Persicaria perfoliata*

| **Extracts** | **Percentage yield** |
| --- | --- |
| Aqueous extract | 14.09 |
| Methanol extract | 11.18 |
| Ethanol extract | 5.76 |
| Ethyl acetate extract | 3.51 |
| DCM extract | 2.53 |
| Hexane extract | 0.84 |

**Table 2**

Preliminary qualitative phytochemical analysis

| **Phytochemicals** | **Crude extracts** |
| --- | --- |
| Alkaloids | + |
| Carbohydrates | + |
| Reducing sugars | + |
| Glycosides | + |
| Amino acids | + |
| Flavonoids | + |
| Phenols | + |
| Tannins | + |
| Terpenoids | + |
| Anthraquinones | + |
| Phytosterols | + |

+ indicates the presence of phytochemicals

**Table 3**

TPC and TFC in different solvent extracts of *P. perfoliata*

| **Extracts** | **Total phenolic content**  **(mg GAE/g)** | **Total flavonoid content (mg QE/g)** |
| --- | --- | --- |
| Aqueous extract | 52.17 ± 3.66^a, b^ | 17.51 ± 3.02^a, b^ |
| Methanol extract | 68.61 ± 0.57^c^ | 40.69 ± 5.0 |
| Ethanol extract | 60.04 ± 6.62^b, c^ | 22.93 ± 6.3^a^ |
| Ethyl acetate extract | 43.36 ± 3.71^a^ | 7.81 ± 1.98^b, c^ |
| DCM extract | 31.07 ± 5.85^d^ | 5.54 ± 2.27^c^ |
| Hexane extract | 26.35 ± 2.76^d^ | 11.6 ± 3.28^b, c^ |

Values followed by different letters or no letters are significantly different from each other at p < 0.05.

**Table 4**

IC_50_ values for plant extracts and nanoparticles against DPPH and α-amylase inhibition

| **Extracts** | **IC_50_ (µg/mL)** | |
| --- | --- | --- |
|  | **Antioxidant activity** | **α-amylase inhibition** |
| Aqueous extract | 452.5 ± 2.82 | 1188.83 ± 33.52 |
| Methanol extract | 185.5 ± 0.99 | - |
| Ethanol extract | 123.1 ± 1.03 | - |
| Ethyl acetate extract | 3036±0.00 | - |
| DCM extract | 116.5 ± 1.63 | - |
| Hexane extract | 69.40 ± 0.13 | - |
| Ag NPs | 144.5 ± 1.36 | 1369.30 ± 46.86 |
| *Quercetin | 3.43 ± 1.61 | - |

* Positive control. – Values not measured. Values are significantly different from each other at p < 0.05.

**Table 5**

ZOI showed by various extracts of *P. perfoliata* and nanoparticles against test organisms

| **Solvent extract** | **Zone of inhibition (mm)** | | | |
| --- | --- | --- | --- | --- |
|  | ***K. pneumoniae*** | ***S. aureus*** | ***E. coli*** | ***S. sonnei*** |
| Aqueous extract | 11 | 9 | 9 | 9 |
| Methanol extract | 9 | 9 | 9 | 11 |
| Ethanol extract | 9 | 9 | 9 | 10 |
| Ethyl acetate extract | 9 | 9 | 9 | 9 |
| Hexane extract | 9 | 9 | 9 | 9 |
| Ag NPs | 16 | 9 | 9 | 9 |
| *Neomycin | 25 | 20 | 23 | 20 |

* Positive control

**Table 6**

MIC and MBC of plant extracts and Ag NPs

| **Extracts** | ***Klebsiella pneumoniae*** | | ***Staphylococcus aureus*** | |
| --- | --- | --- | --- | --- |
|  | **MIC (mg/mL)** | **MBC (mg/mL)** | **MIC (mg/mL)** | **MBC (mg/mL)** |
| Methanol extract | 1.5625 | 3.125 | 0.78125 | 1.5625 |
| Ag NPs | 3.125 | 6.25 | 0.78125 | 1.5625 |
| Aqueous extract | 6.25 | 12.5 | 12.5 | 25 |
| *Neomycin | 0.0039 | 0.0078 | 0.0078 | 0.0156 |

* Positive control.

**Table 7**

Composition of different elements in the synthesized Ag NPs

| **Element** | **Weight %** | **Atomic %** |
| --- | --- | --- |
| C K | 112 | 46.3 |
| N K | 1.7 | 5.9 |
| O K | 2.9 | 9.1 |
| Ag L | 84.2 | 38.8 |

**Table 8**

LC_50_ of silver nanoparticles and aqueous extract of *P. perfoliate*

| **Samples** | **LC_50_ (μg/mL)** |
| --- | --- |
| Ag NPs | 3486.55 ± 1378.94 |
| Aqueous extract | 1873.54 ± 543.10 |

Values are not significantly different from each other at p > 0.05.

**Table 9**

IC_50_ values of silver nanoparticles against HeLa and A549 cell lines

| **Cell lines** | **IC_50_ (μg/mL)** |
| --- | --- |
| HeLa | 251.86 ± 58.90 |
| A549 | 1350.12 ± 1644.09 |

Values are not significantly different from each other at p > 0.05.
